# Supplementary material for: Genetic structure of marine and lake forms of Pacific herring Clupea pallasii
Source: PeerJ. 2021 Nov 2;9:e12444. doi: 10.7717/peerj.12444 (PMC8570158; doi:10.7717/peerj.12444)
Supplement: Supplemental Information 1 [file peerj-09-12444-s001.docx]

**Supplementary Material for**

**Genetic structure of marine and freshwater forms of Pacific herring *Clupea pallasii***

Svetlana Yu. Orlova ^1,2*^, Sergey M. Rastorguev ^3^, Tatyana A. Bagno ^3^, Denis E. Kurnosov ^4^, Artem Nedoluzhko ^5^

*^1^ Russian Federal Research Institute of Fisheries and Oceanography, Moscow, 107140, Russia.*

*^2^ Shirshov Institute of Oceanology of Russian Academy of Sciences, Moscow, 117997, Russia*

*^3^ National Research Center “Kurchatov Institute”, Moscow, 123182, Russia*

*^4^ Russian Federal Research Institute of Fisheries and Oceanography, Pacific Branch (TINRO), Vladivostok, 690091, Russia*

*^5^ Faculty of Biosciences and Aquaculture, Nord University, Bodø, 8049, Norway*

* Corresponding authors: Svetlana Yu. Orlova: [kordicheva@rambler.ru](mailto:kordicheva@rambler.ru); Artem Nedoluzhko: [artem.nedoluzhko@nord.no](mailto:artem.nedoluzhko@nord.no)

**Supplementary Table 1**. Illumina generated reads and number of mapped reads to Atlantic herring (*Clupea harengus*) reference genome sequence (Ch_v2.0.2)

| Population name | Library name | Total number of reads | Total number of mapped reads | Percentage of mapped reads | NCBI accession number |
| --- | --- | --- | --- | --- | --- |
| Scva | cva62_4_7 | 5,666,616 | 4,738,602 | 83.62% | SAMN18506977 |
|  | cva64_5_7 | 3,018,548 | 2,509,976 | 83.15% | SAMN18506978 |
|  | cva65_6_8 | 6,021,332 | 4,904,522 | 81.45% | SAMN18506979 |
| Scvk | cvk62_4_8 | 11,185,124 | 7,857,452 | 70.25% | SAMN18506980 |
|  | cvk64_5_8 | 7,580,228 | 6,449,110 | 85.08% | SAMN18506981 |
|  | cvk65_6_9 | 4,541,732 | 3,357,444 | 73.92% | SAMN18506982 |
| Fain | Fain_1_1 | 2,647,206 | 2,231,822 | 84.31% | SAMN18506983 |
|  | Fain_1_2 | 3,998,342 | 3,460,126 | 86.54% | SAMN18506984 |
|  | Fain_1_3 | 3,418,226 | 2,869,777 | 83.96% | SAMN18506985 |
|  | Fain_1_4 | 5,600,290 | 4,877,823 | 87.10% | SAMN18506986 |
|  | Fain_1_5 | 3,033,190 | 2,589,456 | 85.37% | SAMN18506987 |
| Fnerp | Fnerp_3_1 | 10,390,416 | 9,030,416 | 86.91% | SAMN18506988 |
|  | Fnerp_3_2 | 9,659,486 | 8,452,136 | 87.50% | SAMN18506989 |
|  | Fnerp_3_3 | 10,079,748 | 8,787,378 | 87.18% | SAMN18506990 |
|  | Fnerp_3_4 | 7,468,048 | 6,607,955 | 88.48% | SAMN18506991 |
|  | Fnerp_3_5 | 1,853,794 | 1,451,606 | 78.30% | SAMN18506992 |
| Fvill | Fvill_2_1 | 3,619,684 | 3,097,004 | 85.56% | SAMN18506993 |
|  | Fvill_2_2 | 4,001,572 | 3,358,642 | 83.93% | SAMN18506994 |
|  | Fvill_2_3 | 2,572,408 | 2,155,311 | 83.79% | SAMN18506995 |
|  | Fvill_2_4 | 3,656,196 | 3,136,376 | 85.78% | SAMN18506996 |
|  | Fvill_2_5 | 4,601,738 | 3,951,322 | 85.87% | SAMN18506997 |
| Salex | Salex_4_1 | 5,179,860 | 4,543,173 | 87.71% | SAMN18506998 |
|  | Salex_4_2 | 6,097,712 | 5,300,285 | 86.92% | SAMN18506999 |
|  | Salex_4_3 | 6,324,602 | 5,488,450 | 86.78% | SAMN18507000 |
|  | Salex_4_4 | 4,694,576 | 4,056,387 | 86.41% | SAMN18507001 |
|  | Salex_4_5 | 12,271,572 | 10,390,156 | 84.67% | SAMN18507002 |
| Samur | Samur_5_1 | 2,908,232 | 2,495,497 | 85.81% | SAMN18507003 |
|  | Samur_5_2 | 4,198,660 | 3,577,821 | 85.21% | SAMN18507004 |
|  | Samur_5_3 | 3,673,618 | 3,140,279 | 85.48% | SAMN18507005 |
|  | Samur_5_4 | 4,625,562 | 4,022,794 | 86.97% | SAMN18507006 |
|  | Samur_5_5 | 11,583,482 | 9,801,069 | 84.61% | SAMN18507007 |
| Seve | Seve_7_1 | 3,133,708 | 2,745,338 | 87.61% | SAMN18507008 |
|  | Seve_7_2 | 2,941,022 | 2,524,902 | 85.85% | SAMN18507009 |
|  | Seve_7_3 | 4,041,428 | 3,487,819 | 86.30% | SAMN18507010 |
|  | Seve_7_4 | 3,921,344 | 3,427,086 | 87.40% | SAMN18507011 |
|  | Seve_7_5 | 1,946,010 | 1,688,332 | 86.76% | SAMN18507012 |
| Sk12 | Sk12_8_1 | 1,367,174 | 1,162,808 | 85.05% | SAMN18507013 |
|  | Sk12_8_2 | 1,490,228 | 1,259,581 | 84.52% | SAMN18507014 |
|  | Sk12_8_3 | 2,122,210 | 1,819,568 | 85.74% | SAMN18507015 |
|  | Sk12_8_4 | 1,501,542 | 1,291,062 | 85.98% | SAMN18507016 |
|  | Sk12_8_5 | 46,952 | 28,130 | 59.91% | SAMN18507017 |
| Skrg | Skrg_6_1 | 2,967,480 | 2,593,525 | 87.40% | SAMN18507018 |
|  | Skrg_6_2 | 567,752 | 468,851 | 82.58% | SAMN18507019 |
|  | Skrg_6_3 | 2,313,992 | 1,990,373 | 86.01% | SAMN18507020 |
|  | Skrg_6_4 | 2,354,066 | 2,059,382 | 87.48% | SAMN18507021 |
|  | Skrg_6_5 | 1,326,658 | 997,854 | 75.22% | SAMN18507022 |
| Sukur | Sukur_9_1 | 611,838 | 503,972 | 82.37% | SAMN18507023 |
|  | Sukur_9_2 | 655,364 | 550,188 | 83.95% | SAMN18507024 |
|  | Sukur_9_3 | 2,457,912 | 2,092,991 | 85.15% | SAMN18507025 |
|  | Sukur_9_4 | 54,746 | 34,742 | 63.46% | SAMN18507026 |
|  | Sukur_9_5 | 433,684 | 343,676 | 79.25% | SAMN18507027 |
| Sclu7 | clu738_5_1 | 1,622,878 | 1,019,335 | 62.81% | SAMN18507028 |
|  | clu739_6_1 | 1,056,650 | 698,535 | 66.11% | SAMN18507029 |
|  | clu743_7_1 | 1,327,108 | 876,993 | 66.08% | SAMN18507030 |
|  | Total | 216,433,546 | 182,355,210 | 82.62% |  |
